# Establishment of a novel *in vitro* model of stratified epithelial wound healing with barrier function

**DOI:** 10.1038/srep19395

**Published:** 2016-01-13

**Authors:** Miguel Gonzalez-Andrades, Luis Alonso-Pastor, Jérôme Mauris, Andrea Cruzat, Claes H. Dohlman, Pablo Argüeso

**Affiliations:** 1 Schepens Eye Research Institute and Massachusetts Eye and Ear, Department of Ophthalmology, Harvard Medical School, Boston, Massachusetts, USA; 2 Changing Places Research Group, Media Lab, Massachusetts Institute of Technology, Cambridge, Massachusetts, USA

## Abstract

The repair of wounds through collective movement of epithelial cells is a fundamental process in multicellular organisms. In stratified epithelia such as the cornea and skin, healing occurs in three steps that include a latent, migratory, and reconstruction phases. Several simple and inexpensive assays have been developed to study the biology of cell migration *in vitro.* However, these assays are mostly based on monolayer systems that fail to reproduce the differentiation processes associated to multilayered systems. Here, we describe a straightforward *in vitro* wound assay to evaluate the healing and restoration of barrier function in stratified human corneal epithelial cells. In this assay, circular punch injuries lead to the collective migration of the epithelium as coherent sheets. The closure of the wound was associated with the restoration of the transcellular barrier and the re-establishment of apical intercellular junctions. Altogether, this new model of wound healing provides an important research tool to study the mechanisms leading to barrier function in stratified epithelia and may facilitate the development of future therapeutic applications.

Re-epithelialization is an essential biological process critical to restore an intact barrier in organ systems such as the cornea, skin and gastrointestinal tract following a wound^1^. Defects in this function are associated with impaired or delayed wound healing and the appearance of persistent epithelial defects that have proven painful and difficult to treat. In cornea, most epithelial wounds are repaired promptly. However, under certain clinical conditions, such as herpes simplex virus infection, neurotrophic keratopathy, or diabetic keratopathy, the epithelial defects can persist despite conventional treatment, leading to major complications such as corneal melting, perforation and ultimately loss of vision^2^. Elucidating the molecular mechanisms that underlie the proper closure of corneal wounds is important for the development of novel treatments to promote healing and to the clinical management of epithelial lesions.

Numerous models and methods of injury have been described to study the migratory process in corneal epithelial cells^3^. The animal models present the advantage of having a complex environment that is spatially organized and includes interactions at the systemic level. However, they are somehow limited by ethical concerns, high costs, and uncertain extension of the results to human wound healing^4^,^5^. Human corneal *ex vivo* models have been developed, but their use has been hampered by the difficulty of obtaining healthy human donor tissue and poor standardization due to cornea-to-cornea variability^6^,^7^. The use of *in vitro* models can simplify the characterization of specific biological processes that are difficult to study *in vivo* because of their complexity. For this purpose, tissue engineered scaffolds have been developed, but are expensive and difficult to produce and manipulate^3^. Culture models using human corneal epithelial cell lines, on the other hand, offer a series of advantages such as simplicity, high level of reproducibility and relatively low costs^3^,^8^.

Similarly to the skin, the barrier properties of the cornea are to a great extent provided by a stratified epithelium that functions to protect the surface from physical and chemical agents. One of the first repair responses that follow physical abrasion is the rearrangement of individual cells from the stratified epithelium adjacent to the wound^2^,^9^. In this phase, also known as latent phase, the wound edge shows no obvious movement. Then, during the migratory phase, epithelial cells from different layers, and from up to 1 mm back from the edge, migrate as a coherent unit with very similar speeds to close the wound^2^,^10^. This is followed by a reconstruction phase, when cells mostly change morphology and become flattened^10^. This phase of wound repair is essential for the reestablishment of the normal appearance of the epithelia and the restoration of barrier function^11^. Unfortunately, most culture models evaluating migration of cells from stratified tissues are based on monolayer cultures, which fail to reproduce the three phases of epithelial healing. The aim of this study was to develop and characterize a simple and reproducible *in vitro* model of wound healing that could potentially be used to study the different phases of healing in stratified epithelium.

## Results

### Morphometric analysis of epithelial wounds

**Figure 1:**
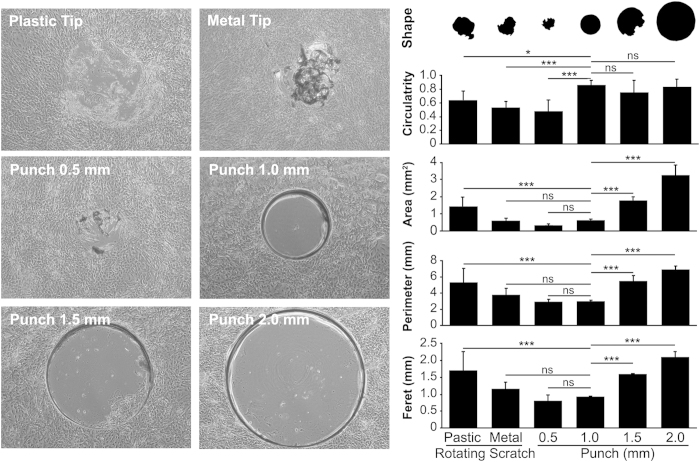
Morphometric analysis of epithelial wounds. Stratified cultures of human corneal epithelial cells were injured by rotation using a plastic or metal tip (rotating scratch model), or using dermal punchs (0.5, 1.0, 1.5 or 2.0 mm diameter) followed by epithelial debridement (punch model). Morphology of the injured area was analyzed by measuring main parameters of the wound shape, including circularity, area, perimeter and Feret diameter. The area and perimeter of the wounds ranged from 0.3 to 3.3 mm^2^ and 2.9 to 7.0 mm, respectively. The values for circularity and Feret diameter ranged from 0.48 to 0.87 and 0.8 to 2.1 mm, respectively. Use of plastic or metal tips produced modest results in terms of shape and circularity. Punch injuries followed by epithelial debridement using a disposable plastic tip (i.e., 1.0, 1.5 and 2.0 mm punch) produced consistent shapes and high circularity values. Results are displayed as mean +/− SD (N = 9). Significance was determined using one-way ANOVA with Bonferroni’s post-hoc test. *p < 0.05; ***p < 0.001. ns, non-significant.

**Figure 2:**
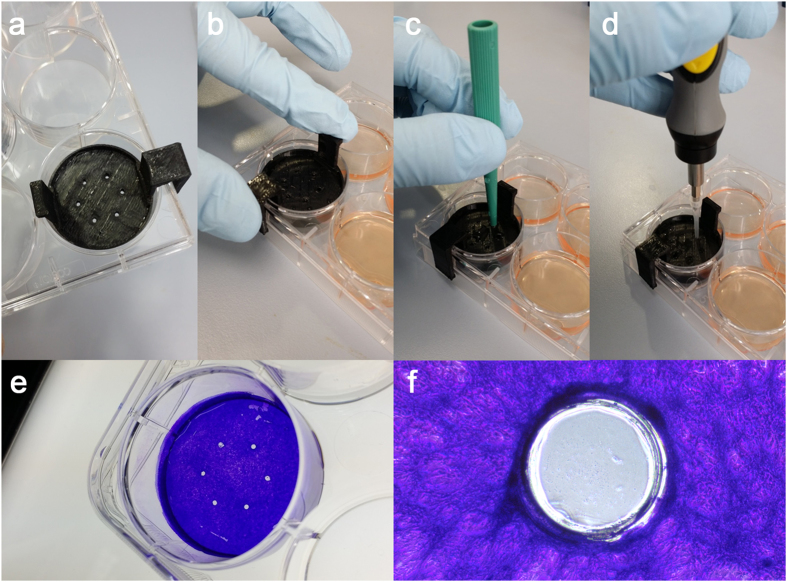
Procedure to generate multiple wounds using a 3D-printed punch template. (**a**) Circular wounds of 1 mm in diameter were made using a punch template in areas equidistant to the center of the culture. (**b–d**) The medium was removed before inserting the template into the culture well. Wounds were produced by pressing and rotating the metal cutting edge of the punch. Cells within the wound were debrided by rotation of a 10 μl disposable tip connected to a cordless precision screwdriver. (**e–f**) The circular wounds can be observed after staining with crystal violet.

Understanding how re-epithelialization and induction of barrier function are orchestrated in multilayered wound healing models requires the development of injury assays that can be systematically and quantitatively reproduced^12^. Our initial attempts to create *in vitro* wounds in stratified epithelial cells were based on the commonly used scratch-induced directional wound healing assay developed for cell monolayers^13^. However, use of this technique in stratified cultures resulted in the detachment of the epithelial sheet from the plastic surface in areas adjacent to the wound and the formation of irregular wounds. Therefore, in subsequent experiments, we used rotation to facilitate the sectioning of the epithelial sheet. Analyses of the wound morphology revealed that use of plastic or metal tips in rotating scratch injury models produced modest results in terms of shape and circularity (Fig. 1). On the other hand, punch injuries, particularly those followed by epithelial debridement using a disposable plastic tip (i.e., 1.0, 1.5 and 2.0 mm punch), produced consistent shapes that were associated with high circularity values. The reproducibility and uniformity of these injuries was dependent on the application of comparable pressure on the punch and the generation of a concentric incision groove in the tissue culture plate around the punch wound. In this injury model, the use of a 3D-printed punch template enabled the plastic tip to be confined within the small wound area produced by the 1.0 mm punch and also facilitated the generation of multiple wounds within a single tissue culture well (Fig. 2).

### Promotion of wound closure

**Figure 3:**
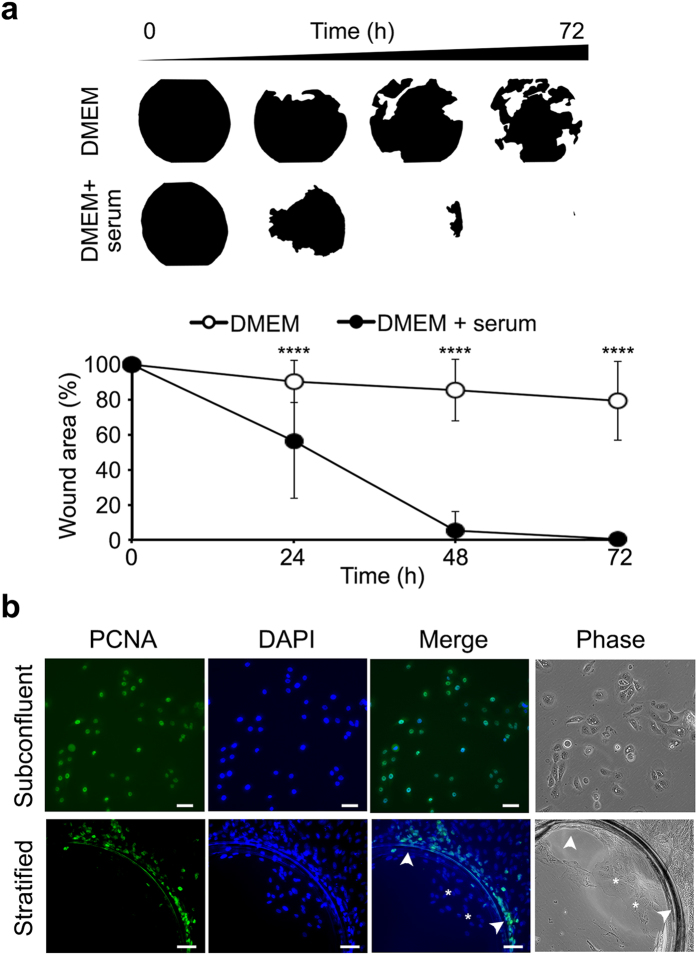
Serum promotes re-epithelialization and wound closure. Circular wounds of 1 mm in diameter were made using a punch template. (**a**) Morphometric analysis demonstrated that the extent of re-epithelialization was enhanced in the presence of culture media containing serum. In the absence of serum, none of the wounds was able to completely re-epithelialize at 72 h, whereas in the presence of serum, the wounded area was significantly reduced at 48 h and completely closed at 72 h. Results are displayed as mean +/− SD (N = 18). Significance was determined using the Mann-Whitney test. ****p < 0.0001. (**b**) In control experiments, the presence of proliferative activity was demonstrated by staining for PCNA (green) in subconfluent cultures of human corneal epithelial cells. Following injury to stratified cultures, clusters of PCNA-positive cells appeared within the concentric incision groove around the punch wound (arrowheads), but not in actively migrating cells (asterisks). Nuclei were counterstained using DAPI (blue). Scale bar, 100 μm.

Injury to a stratified epithelium initiates a cascade of events that includes rearrangement of individual cells and the initiation of a migratory phase to close the wound. This phase is dependent on chemotactic cytokines and mitogenic growth factors^14^,^15^. Along with these factors, *in vitro* studies have supported a critical role for serum in promoting cell viability, proliferation and migration of epithelial cells^16^. In our experiments, the wound closure was significantly promoted following addition of serum to the cell culture media (Fig. 3a). Here, the wounded area was re-epithelialized by more than 40% at 24 h and 90% at 48 h, and a complete closed wound was observed at 72 h. The presence of proliferative activity following addition of serum was further demonstrated by staining for proliferating cell nuclear antigen or PCNA, a marker of growing cells entering the early S phase of the cell cycle^17^,^18^. Clusters of PCNA-positive cells appeared within the concentric incision groove around the punch wound, but not in actively migrating cells, a phenomenon known as migration-proliferation dichotomy (Fig. 3b). It is worth to note that wounds in these experiments were carried out in areas equidistant to the center of the well in the culture plate, since differences in re-epithelialization rates were observed depending on whether the wounds were performed towards the periphery or center of the well.

**Figure 4:**
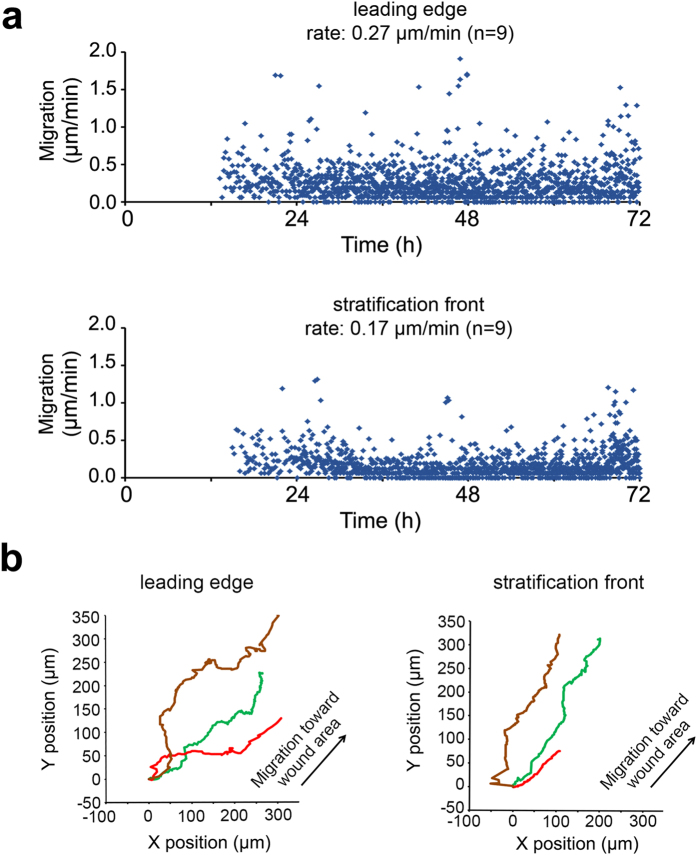
Wound healing involves migration by unified sliding of cells at the leading edge and stratification front. Circular wounds of 1 mm in diameter were made using a punch template. The migration of individual human corneal epithelial cells in stratified cultures and in the presence of serum was analyzed by time-lapse imaging. **(a)** Cell-tracking experiments revealed that cells at the leading edge and stratification front migrated with an average trajectory speed of 0.27 and 0.17 μm/min respectively. **(b)** Following a wound, epithelial cells at the leading edge and stratification front healed by unified directional movement towards the wound area. Representative cell tracks in three wounds are shown. Tracks are color-coded and represent individual wounds.

Direct visualization of the migratory process by time-lapse microscopy revealed an initial accumulation of cell mass within the incision groove in the tissue culture plate followed by unified sliding of the healing epithelium towards the wound (Supplementary Movie 1). During this migratory phase, most cells at the leading edge remained at the wound edge and were not actively replaced by other cells. Cell-tracking experiments showed that the average rate of migration of cells at the leading edge during this phase was 0.27 μm/min (Fig. 4a). Cells at the stratification front moved behind the leading edge at a slower average rate of 0.17 μm/min. Both the leading edge and the stratification front maintained similar trajectories and relative position (Fig. 4b).

### Restoration of barrier function

**Figure 5:**
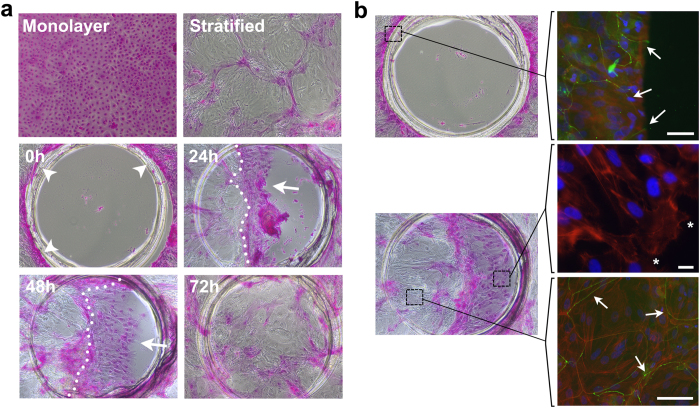
Barrier function is restored as epithelial cells migrate en bloc. **(a)** Circular wounds of 1 mm in diameter were made using a punch template. The extent of rose bengal dye penetrance was assessed at 0, 24, 48 and 72 h following culture in serum-containing medium. Induction of stratification in control cultures resulted in the formation of islands of stratified cells that excluded the dye. A defined staining of the wound margins was observed immediately after wounding (arrowheads). Epithelial migration was characterized by uptake of dye by cells at the leading edge (arrows) but not behind the stratification front (dotted line). Closure of the wound at 72 h was associated with the complete restoration of barrier function. **(b)** For immunofluorescence microcopy, stratified cultures in chambered slides were wounded using a 1.5 mm punch and manually scraped using a pipette tip. Occludin (green) was detected at the cellular margin after wounding (arrows), and was restored to the intercellular junctions within the stratification front during migration. Cells at the leading edge (asterisks) extended lamellipodial protrusions as shown by actin staining (red) and were characterized by lack of occludin localization at the cell-cell junction. Nuclei were counterstained using DAPI (blue). Scale bars, 50 μm (top), 20 μm (middle), 100 μm (bottom).

Restoration of the transcellular and paracellular barrier function in *in vitro* models of wound healing is critical to understanding the biological processes associated with the reconstruction phase of healing. To determine whether wound closure was associated with restoration of transcellular barrier function in our model of stratified epithelial wound healing, we took advantage of the rose bengal penetration assay. In this assay, uptake of rose bengal by the cell culture is dependent on the character of the apical glycocalyx and the ability to synthesize cell surface mucins^19^,^20^,^21^,^22^. The presence of a fully functional glycocalyx barrier protects against rose bengal uptake, whereas positive staining of the epithelia indicates the presence of a compromised transcellular barrier. As shown in Fig. 5, monolayer cultures of corneal epithelial cells stained positively with rose bengal. In contrast, stratified cultures were characterized by the presence of islands of stratified cells that excluded the dye. Analyses of the stratified culture immediately after wounding (t = 0 h) resulted in a defined staining of the wound margins, indicative of epithelial damage induced by the cutting edge of the punch. Subsequent to the initial wound, cell migration was characterized by the presence of a leading edge of monolayer cells that stained positively for rose bengal, and a stratified zone behind the edge that contained areas of rose bengal exclusion. This stratified zone was observed within the wound at 24 and 48 h, and mostly covered the entire wound at 72 h.

In addition to the transcellular barrier, the formation of tight junctions is crucial for the establishment of paracellular barrier function in polarized epithelial sheets. Analyses of stratified cultures immediately after wounding revealed abrupt interruption of the tight junction-associated protein occludin at the wound margin (Fig. 5b, upper panel). During the migratory phase, epithelial cells at the leading edge of the migrating sheet extended lamellipodial protrusions and were characterized by lack of occludin localization at the cell-cell junction (Fig. 5b, lower panel). However, occludin expression was restored to apical intercellular junctions within the stratification front behind the leading edge, indicating that tight junctions are immediately formed after the epithelium becomes multilayered, and suggesting the presence of a reconstruction phase and the normalization of the barrier function of the epithelial sheet during wound closure.

## Discussion

Understanding the mechanisms that lead to restoration of barrier function in stratified epithelia during wound healing is essential to maintain tissue integrity and reduce complications such as infection and corneal melting. The culture of epithelial cells in monolayers is useful as a straightforward method to measure cell migration *in vitro*, but is limited in capturing the biological processes and higher level of tissue control that characterize the reconstruction phase of wound closure in stratified epithelia^12^. Here, we have established a simple and inexpensive *in vitro* model of wound healing that displays features associated with the re-epithelialization of stratified epithelia that include unified sliding of cell sheets and restoration of epithelial barrier function.

Our model allows the study of healing in a multilayered system that mimics the characteristics of the native human corneal epithelium in terms of stratification and barrier function. The cell line used in our experiments was derived by sequential transduction to express a dominant negative p53 protein, a p16^INK4A^-resistant point mutant of cdk4, and the catalytic subunit of telomerase, which enabled the cells to bypass senescence^23^. When grown on plastic in the presence of high-calcium medium and serum, the cultures are known to exhibit multiple cell layers and expression of fully glycosylated transmembrane mucins on the apical cell layer^22^,^23^. Other immortalized cell lines (e.g., HCjE, hTCEpi) have also been shown to stratify and differentiate similar to normal epithelium^23^,^24^ and could potentially be used for the evaluation of wound healing in stratified culture conditions. In our experiments, wounding of the stratified epithelium with a punch initiated a migratory phase that was dependent on the culture media employed. We found that medium with serum was crucial to promote re-epithelialization in the stratified cultures, in agreement with previous findings reporting successful use of autologous serum in promoting healing during the clinical management of persistent corneal epithelial defects^25^,^26^. Further, migration was associated with the presence of active proliferative cells within the concentric incision groove around the punch wound, in accordance with previous data showing an abundance of proliferative corneal epithelial cells in micro-ridges of engineered constructs^27^. The presence of proliferating cells behind the leading edge may provide a mass of cells ready to migrate and stratify^28^,^29^.

In our setting, the selection and consistency of the pressure applied to the punch (300–450 g/mm^2^) was critical to maintain the reproducibility and the re-epithelization rate of the injury. Higher pressures significantly altered the topography of the plastic surface and resulted in the formation of grooves that drastically impaired the migration of the cell sheet across the wound margin (data not shown). Elegant studies by Dalton *et al.* have demonstrated that migrating epithelia conforms to the contours of indentations and that cell movement across microgrooves is impaired in a manner that is dependent on the depth of the indentation^30^. Although not within the scope of our current study, the design of automatic control systems to refine the constant and uniform application of pressure to the punch will prove crucial to enhance the standardization of the procedure, particularly for studies requiring high throughput analyses.

Two explanations have been traditionally proposed to describe how stratified epithelia heals following a wound; the first involves migration (“sliding”) of the epithelium as a coherent sheet, and the second crawling (“leap frog”) of individual cells over each other^31^. Although multiple factors, such as wound size and environment, may affect the migrating behavior of the leading cells, there is growing evidence supporting a mechanism by which epithelial cells migrate collectively as coherent sheets^10^,^12^,^32^. The visualization of the migratory process and cell tracking data in our model were consistent with this mechanism, and indicated that the stratified corneal epithelium heals mainly using sliding movements. Further, the average rate of cell migration in our model was comparable to previous data of collective cell-sheet movement reporting migration rates between 0.20 and 0.36 μm/min at the leading edge^10^,^32^, although slightly inferior to those reported *in vivo,* in the order of 0.5–1.0 μm/min^33^. These differences observed in the *in vitro* models could be attributed to lack of extracellular matrix components and growth factors such as those found in the tear fluid, known to influence corneal epithelial wound healing. Future studies in this area will further our understanding of the relative importance of these factors in modulating the rate of cell migration in experimental models using multilayered cultures.

The robust closure of human wounds requires a reconstruction phase in which cells differentiate and a well-layered structure with barrier function is restored, with the presence of a suprabasal compartment with apical-basal polarity and the re-establishment of mature cell junctions^12^,^34^,^35^. Use of the rose bengal dye allowed us to determine that closure of the experimental wound in the stratified cultures was associated with restoration of a functional glycocalyx barrier. Further, we observed apical localization of occludin at the tight junction and rose bengal exclusion as the multilayered epithelium migrated over the wound. Rapid restoration of occludin in apical cells of migrating epithelia of multilayered wounds indicates a mechanically stable suprabasal compartment^12^. On the contrary, junctional loss of occludin is required to promote directed cell migration and to retain the migratory flexibility necessary for collective migration^12^,^36^,^37^,^38^. In support of this concept, we found that migrating cells immediately behind the wound edge were characterized by loss of occludin localization within the intercellular junctions.

In summary, we have developed a simple and inexpensive *in vitro* model of wound healing using an immortalized epithelial cell line grown as a multilayered culture. The method enabled us to obtain reproducible wounds that were associated with the coherent migration of epithelial sheets and the restoration of barrier integrity. This model may prove a useful system to better understanding the factors controlling the different phases of human wound healing in stratified epithelia, and could constitute a valuable tool for preclinical wound healing research.

## Methods

### Cell Culture

Telomerase-immortalized human corneal-limbal epithelial cells were grown in a stratified cell culture system at 37 °C and 5% CO_2_ as previously reported^39^. The ability of the cell line to differentiate in the same way as native tissue has been described elsewhere^22^,^23^,^40^,^41^. Briefly, cells were grown as monolayers in keratinocyte serum-free medium (K-SFM) (Life Technologies; Carlsbad, CA) to achieve confluence. Cells were then incubated in Dulbecco’s modified Eagle’s medium (DMEM)/F-12 (Sigma-Aldrich; St. Louis, MO) supplemented with 10% newborn calf serum (Thermo Scientific; Rockford, IL) and 10 ng/ml EGF (Life Technologies) for 7 days to promote stratification and differentiation. Immediately after wounding, cells were incubated with DMEM/F-12 with or without 10% newborn calf serum.

### Generation of wounds

Two types of corneal epithelial cell injury (rotating scratch and punch) were performed in areas equidistant to the center of the culture in six-well tissue culture plates (Corning Inc.; New York, NY). For the rotating scratch injury, wounds were produced by manually rotating a standard 200 μl plastic pipette tip (1 mm diameter; Eppendorf; Hauppauge, NY) or a 0.5 mm titanium-coated diamond burr (Sona Enterprises, Hangzhou, China) at a 90° angle over the culture for approximately 5 sec. For the punch injury, wounds were produced by pressing (300–450 g/mm^2^) and rotating (180°) the metal cutting edge of a 0.5, 1.0, 1.5 or 2.0 mm Miltex dermal punch (Integra Miltex; York, PA) at a 90-degree angle over the culture for approximately 1 sec. The pressure unit was calculated by dividing the weight applied on the punch by the surface area covered by the punch. For this purpose, a precision digital scale was placed under the tissue culture plate. To maintain sharpness, each punch was only used for approximately 20 wounds. Next, cells within the wound were scraped for approximately 5 sec by rotation of the retractable internal plunger of the punch (0.5 mm punch) or by rotation of a 10 μl disposable SHARP^®^ Precision Barrier Tip (Denville Scientific; South Plainfield, NJ). For the latter, tips were inserted within the punch with the help of a metal rod (1.5 and 2.0 mm punch) or connected to a 100 rpm cordless power precision screwdriver and inserted within the well with the help of a 3D-printed 6-hole punch template (1.0 mm punch). The medium was removed before inserting the template into the culture well. The 3D template was modeled to avoid contact with the cell surface using AutoCAD Mechanical design software (Autodesk; San Rafael, CA). Models were exported as STL-files and processed using ReplicatorG (MakerBot Industries; Brooklyn, NY). 3D printing was carried out on a MakerBot Replicator 2 (MakerBot Industries) using polylactic acid filament.

### Morphometry

Morphometric analyses were performed on phase contrast images of cell cultures immediately after wounding. For phase contrast microscopy, cells in tissue culture plates were wounded and imaged with a 4× objective using an inverted microscope (Nikon Eclipse TS100, Nikon Instruments Inc.; Melville, NY). The perimeter of the wound was manually selected, cropped and converted into a binary (black and white) image. The area, perimeter, Feret diameter (the longest distance between any two points along the selection boundary) and circularity of the image were quantified using the “Analyze Particles” function of ImageJ software (National Institutes of Health; Bethesda, MD; http://rsbweb.nih.gov/ij/). For re-epithelialization studies, cultures incubated with or without serum were imaged with a 10x objective at 0, 24, 48 and 72 h after wounding. The perimeter of the wound was manually selected and the area quantified using the “Measure” function of ImageJ. Per each wound, the ratio of re-epithelialization was normalized to the wounded area at time 0 h.

### Phase Contrast Time-Lapse Microscopy

Phase contrast time-lapse microscopy images were acquired with a 10x objective every 5 minutes over 72 hours using a Leica DMI6000 B microscope (Wetzlar, Germany). Images were analyzed using the particle analysis manual tracking plugin for ImageJ (National Institutes of Health). Progress of wound closure was quantitated every 15 minutes from digital images by tracing the leading edge and the stratification front at three different points in three wounds. Individual paths were plotted, and rates of cell migration and location were determined over time.

### Rose bengal uptake

Transcellular barrier function in cell culture was assayed with the rose bengal anionic dye (Acros Organics; Morris Plains, NJ) as described previously^39^. Cells in tissue culture plates were wounded (1 mm in diameter) using the 3D template. For dye penetrance assay, cells were rinsed with PBS and incubated for 5 min with a 0.1% solution of rose bengal. Afterwards, the dye was aspirated and the culture further washed with PBS. The extent of dye penetrance in cell culture was assessed using an inverted microscope (Nikon Eclipse TS100). Pictures were taken at 10× with a SPOT Insight Fire Wire Camera (Diagnostic Instruments, Inc.; Sterling Heights, MI).

### Immunofluorescence

For staining of proliferating cell nuclear antigen (PCNA), cultures in tissue culture plates were fixed with 4% formaldehyde for 15 min, washed with PBS, and permeabilized with ice-cold methanol for 10 minutes. Following hydration in PBS for 5 minutes, cultures were blocked in PBS containing 3% bovine serum albumin and 0.3% Triton X-100 for 1 h, followed by incubation with a primary anti-PCNA (1:100; Santa Cruz Biotechnology, Inc.; Santa Cruz, CA) antibody overnight at 4 °C. Incubation with the primary antibody was routinely omitted in control experiments. After washing with PBS, the corresponding secondary antibody (Alexa Fluor 488-conjugated goat anti-rabbit IgG; 1:1000; Life Technologies) was applied for 1 hour at room temperature. Culture plates were then washed, mounted in VectaShield mounting medium containing DAPI (Vector Laboratories), and observed on a Zeiss Axio Observer Z1 inverted fluorescent microscope (Carl Zeiss Microimaging GmbH, Jena, Germany).

Double staining of tight junctions and the actin cytoskeleton was performed cells on chambered slides as previously described^42^. Briefly, cultures were fixed in 4% formaldehyde, permeabilized with 1% Triton X-100, and blocked with PBS containing 1% bovine serum albumin for 10 min. Cultures were then incubated in Texas Red-X phalloidin (1:100; Life Technologies) for 1 h at room temperature. Following further permeabilization and blocking, cultures were incubated with a primary anti-occludin (1:100; Life Technologies) antibody overnight at 4 °C and Alexa Fluor 488-conjugated rabbit anti-mouse IgG (1:100; Life Technologies) for 1 hour at room temperature. Slides were mounted in VectaShield mounting medium containing DAPI (Vector Laboratories), and observed on an inverted fluorescent microscope as described above.

### Statistical analyses

Statistical analyses were carried out using one-way ANOVA with Bonferroni’s post-hoc test (Stata/SE statistical software version 12.0; StataCorp; College Station, TX) or Mann-Whitney test (Statview software, Abacus Concepts; Piscataway, NJ). All data are shown as mean +/− SD. In all tests, p-values less than 0.05 were considered statistically significant.

## Additional Information

**How to cite this article**: Gonzalez-Andrades, M. *et al.* Establishment of a novel *in vitro* model of stratified epithelial wound healing with barrier function. *Sci. Rep.* **6**, 19395; doi: 10.1038/srep19395 (2016).

## Supplementary Material

Supplementary Movie 1

Supplementary Information
