# Supplementary material for: Establishment of a novel in vitro model of stratified epithelial wound healing with barrier function
Source: Sci Rep. 2016 Jan 13;6:19395. doi: 10.1038/srep19395 (PMC4725353; doi:10.1038/srep19395)
Supplement: Supplementary Information [file srep19395-s2.doc]

**Establishment of a novel *in vitro* model of stratified epithelial wound healing with barrier function**

*Miguel Gonzalez-Andrades,Luis Alonso-Pastor, Jérôme Mauris, Andrea Cruzat, Claes H. Dohlman and Pablo Argüeso.*

**Supplementary Movie 1.** Phase contrast time-lapse video microscopy of human corneal epithelial cells.
